# Evaluation of short‐term face rejuvenation effects of non‐ablative bipolar radiofrequency treatment performed by Med‐RF^©^ device

**DOI:** 10.1111/srt.13422

**Published:** 2023-08-07

**Authors:** Beniamino Palmieri, Maria Vadala, Valentina Rottigni, Anna Aspiro, Maria Stella Di Carmine, Antonio Scarano

**Affiliations:** ^1^ Department of Medical and Surgical Sciences University of Modena Medical School Modena Italy; ^2^ Second Opinion Medical Network Modena Italy; ^3^ Department of Innovative Technologies in Medicine and Dentistry University of Chieti‐Pescara Chieti Italy

**Keywords:** non‐invasive treatment, radiofrequency, rejuvenation, skin

## Abstract

**Objective:**

Skin laxity and wrinkling are common signs of the aging process. This physiological condition of the skin is often accompanied by psychological and social concerns, especially for females, entailing considerable expenses for cosmetics and pharmaceuticals to cease or slow down its advancement.

The objective of this study was to evaluate the short‐term impact of a new non‐ablative radiofrequency device available in the skin face cosmetic field.

**Methods:**

A randomized study was run on 62 healthy subjects with normal‐age related–grade skin laxity, subdivided into two groups. Group 1 received one treatment with Modula RF device (Wavemed, San Cesareo RM, Italy) over the whole face surface. Group 2 received three monthly treatments.

**Results:**

Clinical improvements of the exposed areas were detected by measuring skin parameters, such as total water content, elasticity, and sebum levels at baseline and 1 h after the treatment. Data showed significant effects of Med‐RF treatment on skin hydration (*p* < 0.001), skin elasticity (*p* < 0.0001), and sebum (*p* = 0.0009). Moreover, a negative linear correlation was obtained between women's age and hydration.

**Conclusion:**

In conclusion, the results of this study suggest that radiofrequency significantly improves the subjective and objective judgment of patients and doctors, supported by the positive results obtained on the skin quality parameters evaluated. These results confirm that Med‐RF technology represents an important tool to achieve face rejuvenation in the field of non‐invasive procedures.

## 1 INTRODUCTION

The dermis represents a skin supporting tissue with an extensive vascular network, neurons, smooth muscle fibers, and fibroblasts, and is mainly composed of collagen (mostly type I), elastin and glycosaminoglycans (GAGs), which are responsible for the resiliency of the skin, its density, tonicity, and elastic texture.^1^, ^2^, ^3^, ^4^

Rhytids (wrinkling) and skin laxity, the primary effects of aging,^5^ are due to several clinical and biological factors.^6^ While the first one is the genetic intrinsic background, promoting slow, irreversible tissue degeneration,^7^ the second one is called extrinsic aging or “photoaging”, which affects the skin as a consequence of chronic exposure to atmospheric agents, mainly ultraviolet radiation^8^, ^9^, ^10^ and oxidative stress.

Collagen loss is considered the most representative histological finding in aged skin.^4^, ^7^, ^11^, ^12^ In fact, collagen fibrils are thickened and wrapped in rope‐like bundles, quite inhomogeneous if compared to younger skin texture.^13^ Elastic fibrils also undergo fragmentation, with a subsequent progressive random cross‐linkage and calcification increase in interfibrillar areas.^2^, ^3^, ^14^, ^15^, ^16^, ^17^, ^18^ In addition, lower levels of collagen are synthetized in vivo and in vitro by aged fibroblasts.

Nowadays, there is a great and widespread demand by patients to reduce the aging and photodamaging appearance by rejuvenation techniques, as a widespread and accepted cosmetic procedure.^19^, ^20^, ^21^ In cosmetic surgery, face surgical liftings have represented the gold standard for skin laxity, with the drawbacks of costs, risks and complications.^22^, ^23^ Alternative to surgery, but also associated with significant side effects (e.g., prolonged and unpleasant post‐treatment downtime), ablative fractional and non‐ablative laser resurfacing have been applied,^5^, ^23^, ^24^, ^25^, ^26^, ^27^, ^28^ as well as cosmetic practices using injectable compounds (fillers).^20^, ^29^, ^30^, ^31^, ^32^, ^33^, ^34^ Then, in recent years, totally non‐invasive procedures have become an appealing option, strongly supported by the market claim of safety and effectiveness .^35^, ^36^

Ablative lasers have been accepted as the gold standard for photodamaged skin rejuvenation for more than half of a decade and many different light sources and light‐based systems have been developed and proven effective to reverse photodamage and age‐associated skin degeneration without epidermal damage.^36^, ^37^, ^38^, ^39^, ^40^

The goal of such non‐ablative devices, based on light or radiofrequency (RF), is to selectively heat the target without injuring the surrounding tissue.^41^ However, laser light can be scattered and diffracted, reducing the delivered energy upon the target as a consequence.^42^ On the contrary, RF devices emit electric waves suitable to any skin type. Low‐intensity and high‐frequency electrical current are recruited to generate heat as a result of tissue resistance to the electron flow.

RF technology has been used in different medical areas such as cardiology, urology, and sleep medicine.^23^, ^25^, ^35^, ^42^, ^43^, ^44^ In dermatology, it has been used for cutting, electrocoagulating (stopping bleeding), cancer overheating, intravenous lumen obliteration, skin rejuvenation, and cellulitis treatment.^23^, ^25^, ^35^, ^45^ RF technologies have been developed accordingly to the physical principle of impedance.

Previous clinical studies showed that a time and temperature combination acts on remodeling and reorientation of collagen bundles with newly formed collagen induction in the follow up.^22^, ^25^, ^36^, ^38^, ^46^, ^47^, ^48^ Different monopolar or bipolar devices have been produced for skin tightening and body contouring,^36^, ^49^ either as do‐it‐yourself techniques or as a medical procedure. Monopolar RF therapy delivers uniform heat at controlled depth to dermal layers, causing direct collagen contraction and immediate skin tightening.^38^, ^50^ In this regard, ThermaCool monopolar system was the very first RF technology introduced in 2001.^46^

A certain number of investigations about cosmetic results on the neck, cheeks, and submandibolar and periorbital laxity showed clinical improvements on a 30%–80% range in the follow‐up 3–6 months.^22^, ^44^, ^47^, ^49^, ^51^, ^52^, ^53^, ^54^, ^55^, ^56^ Bipolar RF devices represent a new and innovative approach to the whole‐body rejuvenation.^25^, ^57^ The hybrid system Accent RF, based on monopolar and bipolar RF, has been reported to have improvements of 56%–75% grade.^58^ A new home‐use RF, that is, TriPollar device for body treatments has been developed by Boisnic and Branchet, who tested dermal fibroblast activity either on experimental human skin model or on patients individual clinical treatments at home.^36^ A significant increase of collagen synthesis by dermal fibroblast activity over a period of 3 months was observed, as well as definite cosmetic improvement of the treated faces.

In the following clinical study, following a simple open design, the short‐term efficacy of a non‐ablative RF treatment used for facial skin rejuvenation practice was assessed on healthy female volunteers. Clinical skin effects were evaluated in terms of hydration, elasticity, and sebum levels before and 1 h after the treatment.

## 2 MATERIALS AND METHODS

### 2.1 Subjects and study design

A total of 62 voluntary, healthy women, aged 40.5 ± 9.60 (mean age ± standard deviation, SD, within a range 23–64 years) were enrolled in the study after informed consent. Negative criteria for inclusion in the study were a history of pacemaker, serious cardiac pathologies, pregnancy, and lactation. The group was divided into two cohorts: the first (named SINGLE group, *n* = 48, mean age 40.39 ± 10.36, range 23–64 years old) was treated once, while the second (named TRIPLE group, *n* = 14, mean age 41.0 ± 7.27, range 31–‐55 years old) was treated with a triple monthly application.

All the subjects received the RF treatment on the whole skin surface of the face for 20 min. Some of the subjects had been using topical support treatment (anti‐age sera, hyaluronic acid, night or day cream) in the previous 6 months, thus the previous use of these treatments was considered in the model by a score ranging from 0 (no treatments) to 4 (all of the treatments).

In both groups, skin quality analysis was tested immediately before and 1 h after the last treatment, monitoring by means of a suitable instrument some parameters such as hydration, elasticity, and sebum levels.

### 2.2 Materials and instrumentation

Treatment was performed by using a non‐ablative bipolar RF device (Modula RF, Wavemed, San Cesareo, RM, Italy), equipped with a bipolar electrode, oscillating its polarity at the frequency of 450‐470 kHz. The energy was delivered by pressing with back‐and‐forth movements, the bipolar tip across the selected skin face areas. Before the treatment, skin moisturizing milk and tonic lotion were applied to clean and prepare the skin to the electric treatment. Then, the area to be treated was covered by conventional ultrasound gel (Eco Supergel, Ceracarta, Italy). The Med‐RF system was used at a relative power % (RP %) ranging from 30% to 50% (maximum power value permitted by the bipolar tip is 9 W). Skin parameters were measured using the Skin Tester System (Selenia, Italy).

Skin Tester is a new device originally planned and validated by our group, including important advancements in comparison with currently available skin analyzers in the dermatological area for diagnostic and qualitative analysis of the face skin. It analyzes several skin parameters to monitor pre‐ and post‐treatment variations.

An ultrasound emitted beam is reflected by the dermal tissues, according to its stromal density and vascular tone.

Furthermore, impedance variation related to intracellular and interstitial water content and photoplethysmography, a reflectometric method to evaluate vascular network dynamics, are included in the diagnostic device. Thus, the following skin parameters are detected:

- total and intracellular water to correctly evaluate hydration;
- extracellular water and water retention to detect tissue fluid engulfment and drain impairment;
- skin pH, related to sebaceous and sweat glands secretion;
- thickness of horny layer;
- sebum epidermic concentration;
- skin elasticity and tonicity parameters, related to elastin, collagen and GAGs imbalance;
- skin reflexology;
- vascular network adaptations.

The Skin tester works and records the operative data through a touch screen display, which has a flat transducer to be applied over the skin surface and requires a gel film to achieve ultrasound delivery.

Analysis results are printed on a ticket with the reference values, so the doctor and patient can save the records of the cosmetic treatment variations.

### 2.3 Statistical analysis

Raw data concerning water content, elasticity, and sebum were analyzed by a multiway repeated measures ANCOVA (GLM procedure, SAS ver. 9.2). The treatment type (SINGLE or TRIPLE) was considered as a fixed effect, the time effect (before/after treatment) was treated as the repeated measure, while the number of support treatments and the age of the women were taken into account as a covariate. ANCOVA results are expressed as mean ± standard error of mean (SEM).

### 2.4 Satisfaction evaluation

At the end of the study, patients’ satisfaction was graded from completely satisfied to completely dissatisfied for the treated area, as shown in Tables 1 and 2.

**TABLE 1:** Patient satisfaction after one treatment (results expressed in percentage).

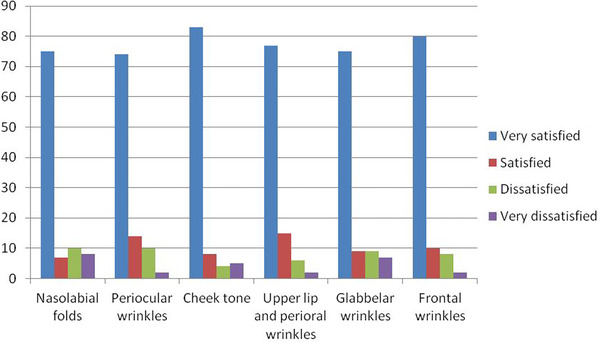

**TABLE 2:** Patient satisfaction after three treatments (results expressed in percentage).

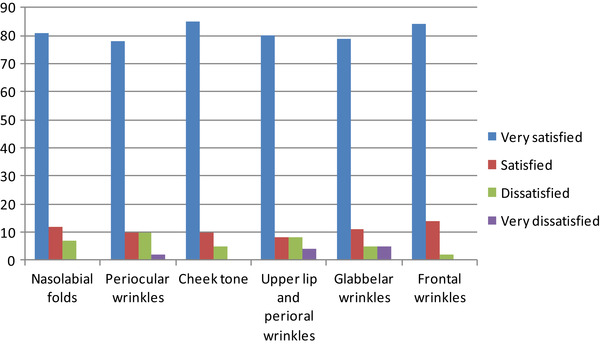

The Doctor Judgment, comparing a standard face photo before and after the treatment, and performing a direct evaluation in the last visit was performed by an independent observer.

The global aesthetic score was, for the specialist doctors, based on the evaluation of the following items, as shown in Table 3:

1. Nasolabial folds: reduced depth and width
2. Periocular wrinkles: reduced number and depth up to disappearance
3. Cheek tone: increased pinch and press elasticity and skin smoothness
4. Upper lip and perioral wrinkles: reduced number and depth
5. Glabellar wrinkles reduced depth
6. Frontal wrinkles: reduced number and depth

**TABLE 3:** Medical specialist average visual subjective score.

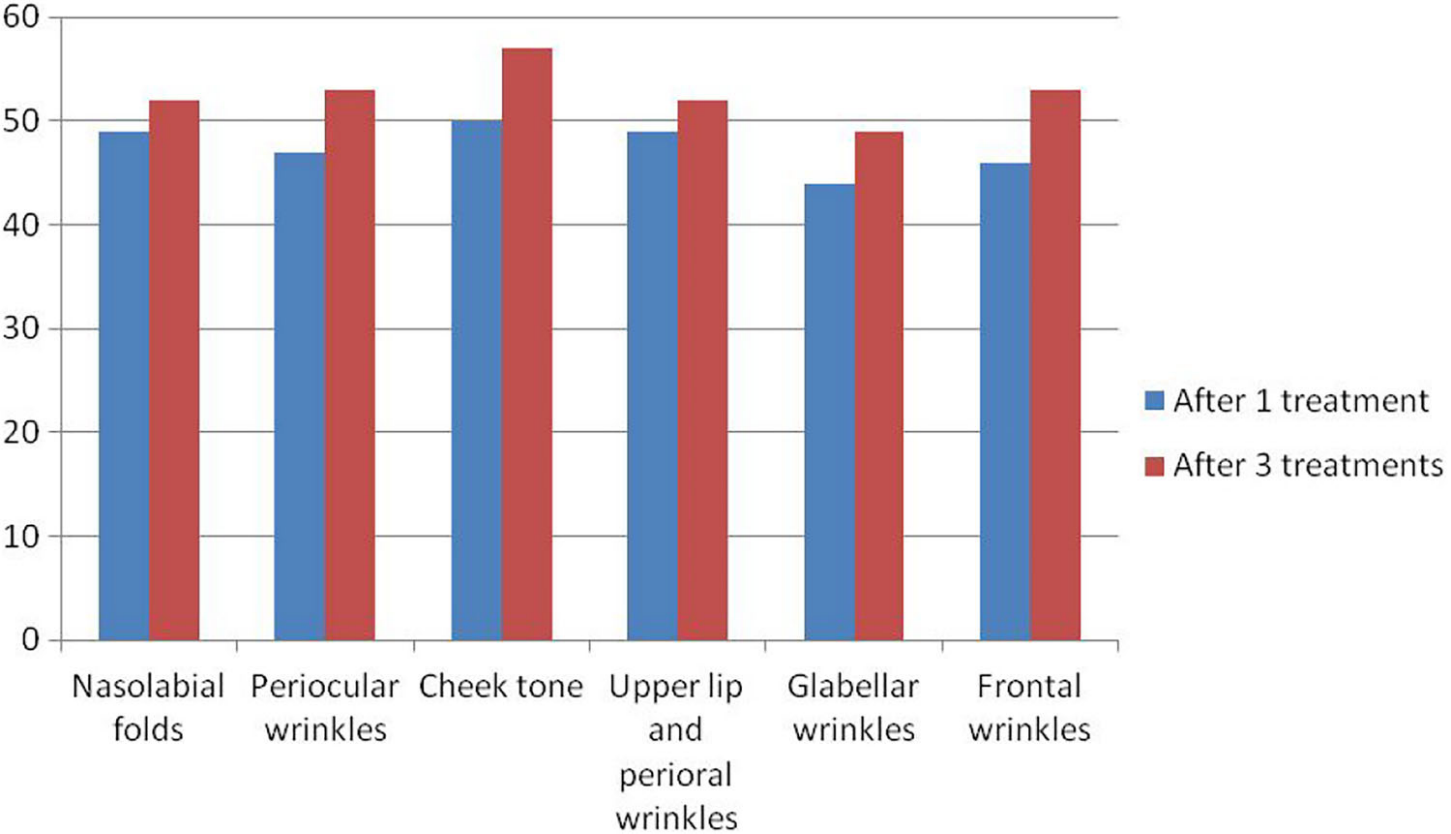

*Note*: A total of three independent specialists scored the results.

**FIGURE 1:**
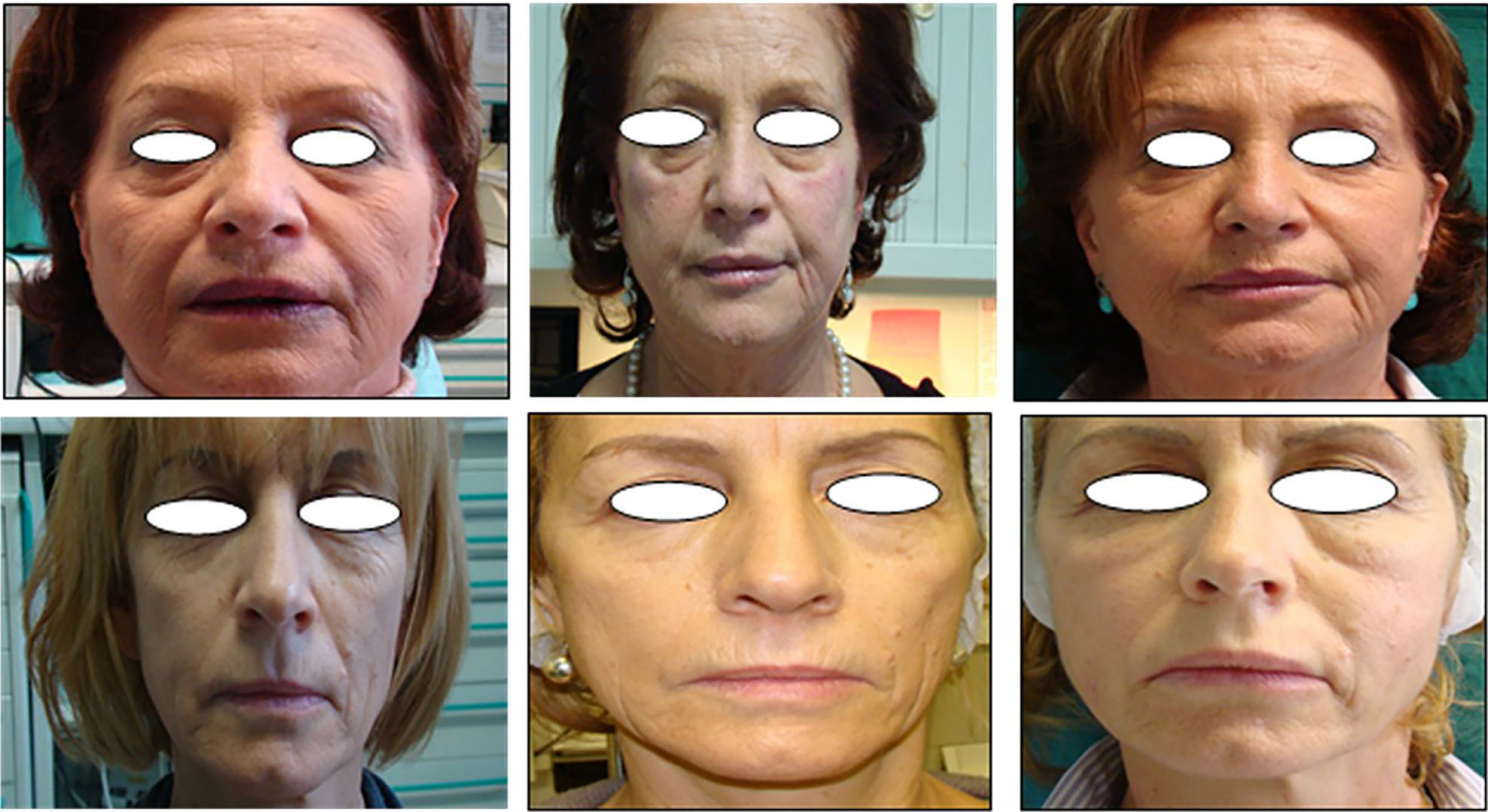
Representative image of two patients, from left to right: after the treatment (baseline); after one treatment, two, and three treatments with RF.

The visual appearance was matched with the skin‐tester evaluation, in order to have an instrumental independent relationship between pre‐ and post‐treatment variations of some skin physicochemical parameters and the sight observation of the clinical effects (Table 4, Figure 1). In addition, the side effects of the treatment were recorded at each follow‐up evaluation.

**TABLE 4:** Skin parameters evaluation pre‐ and post‐treatment for both the single and the triple group.

	Pre‐treatment	Post‐treatment
Total water	50	62
pH	6.1	5.6
Elasticity	37	39
Sebometry	33	40

*Note*: Data refers to the mean of the individual values in arbitrary units (AU).

## 3 RESULTS

### 3.1 More than 70% of the patients declared to be “very satisfied”

As observed in Table 1, more than 70% of the patients declared to be “very satisfied” after one treatment in terms of nasolabial folds, periocular wrinkles, cheek tone, upper lip and perioral wrinkles, glabellar wrinkles and frontal wrinkles. The patients’ satisfaction even increased after three treatments (Figure 1), reaching almost 80% of patients declaring themselves as “very satisfied” (Table 2).

### 3.2 Specialists’ satisfaction of the results stands above 40% on average

As observed in Table 3, the average of satisfaction for the medical specialists in this field stands above 40% after one treatment, increasing after three treatments. Theoretically, the maximal expected outcome of the summarized items scores should have been 60, but the patients’ global judgment was sometimes upgraded due to the improved appeal of the face as a whole as positively appreciated by the patients in their mirror reflections.

### 3.3 Skin parameter's evaluation considerably improved after the treatment

The objective evaluation of the results, performed by evaluating the total water content, the pH of the skin, the elasticity and the sebometry confirmed the subjective previous results, resulting in improved values for all the parameters studied (Table 4). The statistical analysis evidenced a significant effect of RF treatment (*p* < 0.001) on the hydration mean value, which before the treatment was 50.35 ± 2.12 arbitrary units (AU) and after 62.26 ± 1.85 AU. Moreover, a significant difference was observed for the kind of treatment (*p* = 0.03): skin hydration values were 51.08 ± 2.71 AU and 65.13 ± 2.02 AU (SINGLE, before and after treatment, respectively), and 47.86 ± 1.23 AU and 52.43 ± 1.26 AU (TRIPLE, before and after treatment, respectively). The age of the women has a significant effect on hydration (*p* = 0.01), and topical support treatment was borderline significant, with *p* = 0.06.

The statistical analysis evidenced a significant effect of RF treatment (*p* < 0.0001): the elasticity mean value before treatment was 37.0 ± 1.23 arbitrary units (AU) and after 38.4 ± 1.12 AU. Moreover, a significant difference was observed for the kind of treatment (*p* = 0.0001): skin elasticity values were 39.69 ± 1.35 and 40.69 ± 1.26 AU (SINGLE, before and after treatment, respectively), and 27.79 ± 0.63 and 30.64 ± 0.64 (TRIPLE, before and after treatments, respectively). The age of the women has a significant effect on hydration (*p* = 0.0008), and topical support treatment was not statistically significant.

The statistical analysis evidenced a significant effect of RF treatment (*p* = 0.0009): the sebum mean value before treatment was 33.26 ± 2.69 and after 40.42 ± 2.12 (SINGLE, before and after treatment, respectively). The age of the women has a significant effect on sebum (*p* = 0.008), and topical support treatment was statistically significant (*p* = 0.02); no interaction effects were noted between age effect and topical support treatment. Then, the effect of age does not influence the effect on sebum after topical support treatment.

### 3.4 Statistical analysis: age and relations between variables

The distribution of age was normally distributed (Anderson–Darling normality test, *p* > 0.1); the mean age of SINGLE treated women and TRIPLE treated women did not differ (*t*‐test, *p* > 0.05).

In order to evaluate the effect of the treatment depending on age and/or topical support treatment, the regression coefficients of covariates were extrapolated from the models. A negative linear correlation was observed between age and hydration (*b* = −5.45, *p* = 0.044). Older women seem to have a higher number of local support treatments (*b* = 0.67, *p* < 0.001). No interaction effect was noted between age effect and treatment on skin hydration and elasticity, indicating a substantial positive effect independently on the age of the individuals.

## 4 DISCUSSION

The short‐term effects obtained both after a single treatment or after three treatments by bipolar RF device for skin ageing and laxity are described.

The RF administration was well tolerated with very few minor side effects. One case had a moderate vascular active hyperemia with face erythema, lasting less than 3 h, and another one complained of a short‐term paresthesia on the right cheek, the side that had been previously involved in cold incomplete (fully recovered) facial paralysis.

The results suggest that RF significantly improved the skin quality, mainly in terms of total water content, elasticity, and sebum, with a slight increasing trend between pre‐ and 1 h post‐treatment. Furthermore, the increased sebum levels observed after treatment suggest that RF technology restores the microvascular environment and the balance between local lipidic film coating and serum lipidic levels. Total water content (hydration), elasticity, and sebum levels were selected as the most representative indications of beneficial effects.

In fact, total water content indicates the capacity of the skin tissue to be moisturized due to the presence of GAGs (e.g., hyaluronic acid). Optimal percentage values should stand above 50%, has achieved for these patients, especially after three treatments.

Elasticity is referred as the skin resilience under environmental stress inputs, while tonicity (turgor) is related to the skin structural protein content, such as collagen fibrils. Elasticity and tonicity should be as high as possible (>26%), indicating correct levels of hydration, sebum, elastin and collagen content. A recent research showed that RF improves skin laxity and determines gene activation in a panel of proteins that are relevant in the extracellular matrix of dermal connective tissue.^59^ It has been reported that the RF treatment, induces inflammatory reactions starting from immediately after treatment, which triggers the neocollagenesis as a caused by the heat distress protein generation and dermal remodeling.^60^, ^61^, ^62^ So, the RF is a technique that meets the demands of patients seeking minimally invasive methods to remodel adipose tissue and tighten skin. Many clinical studies show the ability of the RF therapy for aesthetic skin tightening of the face and body. Many studies have shown that the RF treatment improves skin elasticity^63^ and improves skin texture and wrinkles.^64^ The results obtained in the present study were above this value, as well as those for sebum levels, which are related to an adequate balance between the skin surface lipidic film defense, microvascular dermic flow, and individual metabolism.

Obviously, the sequence of three RF sessions on a monthly basis achieved more appealing and prolonged cosmetic effects compared with the single treatment, due to a more prolonged intermittent dermal conditioning when the biological effect of the previous RF administration disappears. The rationale for this protocol is supported by histological and ultrastructural studies, in fact the remodeling of collagen bundles with formation of new collagen and expression of messenger RNA for collagen type 1 continues for months after the treatment.^38^, ^50^, ^65^ For this reason, the patients are significantly more satisfied with RF results 6 months after finishing the treatment than 1 month after it, which might be ascribed to induction of fibroblast and continued collagen synthesis.^66^, ^67^

In conclusion, our data confirm that the Med‐RF technology represents an important tool to achieve face rejuvenation in the field of non‐invasive cosmetic procedures, and because of the safety and effectiveness of the treatment, it should be administered as a not invasive cosmetic treatment in the great majority of the ageing population, which is constantly and actively looking for a painless, conservative, and appealing approach.

## CONFLICTS OF INTEREST STATEMENT

The authors declare no conflicts of interest.

## FUNDING INFORMATION

University of Chieti‐Pescara within the CRUI‐CARE Agreement

## ETHICS STATEMENT

The authors confirm that the ethical policies of the journal, as noted on the journal's author guidelines page, have been adhered to. The study was based in the Department of Innovative Technology in Medicine and Dentistry of the University of Chieti‐Pescara in full accordance with ethical principles, including the World Medical Association Declaration of Helsinki (https://www.wma.net/ wp content/uploads/2018/07/DoHOct2008.pdf) and the additional requirements of Italian law. All patients signed informed consent on the adopted procedure.

## Data Availability

The data that support the findings of this study are available on request from the corresponding author. The data are not publicly available due to privacy or ethical restrictions.
